# DNA Methylation Levels of the ACE2 Promoter Are Not Associated with Post-COVID-19 Symptoms in Individuals Who Had Been Hospitalized Due to COVID-19

**DOI:** 10.3390/microorganisms12071304

**Published:** 2024-06-27

**Authors:** César Fernández-de-las-Peñas, Gema Díaz-Gil, Antonio Gil-Crujera, Stella M. Gómez-Sánchez, Silvia Ambite-Quesada, Juan Torres-Macho, Pablo Ryan-Murua, Anabel Franco-Moreno, Oscar J. Pellicer-Valero, Lars Arendt-Nielsen, Rocco Giordano

**Affiliations:** 1 Department of Physical Therapy, Occupational Therapy, Rehabilitation and Physical Medicine, Universidad Rey Juan Carlos, 28922 Alcorcón, Spain; silvia.ambite.quesada@urjc.es; 2 Center for Neuroplasticity and Pain (CNAP), Sensory Motor Interaction (SMI), Department of Health Science and Technology, Faculty of Medicine, Aalborg University, DK 9220 Aalborg, Denmark; lan@hst.aau.dk (L.A.-N.); rg@hst.aau.dk (R.G.); 3 Research Group GAMDES, Department of Basic Health Sciences, Universidad Rey Juan Carlos (URJC), 28922 Alcorcón, Spain; gema.diaz@urjc.es (G.D.-G.); antonio.gil@urjc.es (A.G.-C.); stella.gomez@urjc.es (S.M.G.-S.); 4 Department of Internal Medicine, Hospital Universitario Infanta Leonor-Virgen de la Torre, 28031 Madrid, Spain; juan.torresm@salud.madrid.org (J.T.-M.); pabloryan@gmail.com (P.R.-M.); anaisabel.franco@salud.madrid.org (A.F.-M.); 5 Department of Medicine, School of Medicine, Universidad Complutense de Madrid, 28040 Madrid, Spain; 6 Image Processing Laboratory (IPL), Universitat de València, Parc Científic, 46980 Paterna, Spain; oscar.pellicer@uv.es; 7 Department of Gastroenterology & Hepatology, Mech-Sense, Aalborg University Hospital, DK 9100 Aalborg, Denmark; 8 Steno Diabetes Center North Denmark, Clinical Institute, Aalborg University Hospital, DK 9100 Aalborg, Denmark; 9 Department of Oral and Maxillofacial Surgery, Aalborg University Hospital, DK 9100 Aalborg, Denmark

**Keywords:** methylation, ACE2, post-COVID-19, long COVID

## Abstract

It is known that SARS-CoV-2 can translocate via membrane ACE2 exopeptidase into the host cells, and thus hypomethylation of ACE2 possibly upregulates its expression, enhancing the risk of SARS-CoV-2 infection. This study investigated if DNA methylation levels of the ACE2 promoter are associated with the development of post-COVID-19 symptomatology in a cohort of COVID-19 survivors who had been previously hospitalized. Non-stimulated saliva samples were obtained from 279 (51.5 male, mean age: 56.5 ± 13.0 years old) COVID-19 survivors who were hospitalized during the first wave of the pandemic. A face-to-face interview in which patients described the presence of post-COVID-19 symptoms (defined as a symptom that started no later than three months after SARS-CoV-2 infection) that they suffered from to an experienced healthcare trainer was conducted. Methylation of five CpG dinucleotides in the ACE2 promoter was quantified using bisulfite pyrosequencing. The percentage of methylation (%) was associated with the presence of the following reported post-COVID-19 symptoms: fatigue, dyspnea at rest, dyspnea at exertion, brain fog, memory loss, concentration loss, or gastrointestinal problems. Participants were assessed a mean of 17.8 (SD: 5.3) months after hospitalization. At that time, 88.1% of the patients experienced at least one post-COVID-19 symptom (mean number for each patient: 3.0; SD: 1.9 post-COVID-19 symptoms). Dyspnea at exertion (67.3%), fatigue (62.3%), and memory loss (31.2%) were the most frequent post-COVID-19 symptoms in the sample. Overall, the analysis did not reveal any difference in the methylation of the ACE2 promoter in any of the CpG locations according to the presence or absence of fatigue, dyspnea at rest, dyspnea at exertion, memory loss, brain fog, concentration loss, and gastrointestinal problems. This study did not find an association between methylation of ACE2 promoter and the presence of post-COVID-19 fatigue, dyspnea, cognitive or gastrointestinal problems in previously hospitalized COVID-19 survivors.

## 1. Introduction

The coronavirus disease 2019 (COVID-19) pandemic, caused by the severe acute respiratory syndrome coronavirus 2 (SARS-CoV-2), challenged all healthcare systems around the world and a deeper understanding of the biological mechanisms behind individual responses to the virus was clearly needed. Building on this, the field of epigenetics might provide insights into how COVID-19 induces lasting changes in gene activity, potentially influencing long-term health outcomes. Epigenetics are those molecular processes regulating gene expression without modifying DNA sequence and phenotype and that are influenced by several factors, e.g., environmental exposures, stress, and nutrition [1]. Several epigenetic processes including methylation, histone protein modification, or the action of non-coding RNA (ncRNA) are described [2]. The effects of epigenetic changes induced by SARS-CoV-2 have been of interest from the beginning of the outbreak [3] but are still under investigation with research looking into possible systemic and cellular changes induced by COVID-19 [4]. DNA methylation is an epigenetic mark involved in gene expression regulation, catalyzed by a family of DNA methyltransferases that transfer a methyl group from S-adenyl methionine onto the DNA cytosine to form 5-methylcytosine [5]. In fact, some studies investigating methylation patterns in COVID-19 patients have revealed hypermethylation patterns in interferon-related genes and hypomethylation patterns in inflammatory-associated genes, supporting the presence of a dynamic epigenetic regulation (up or down) of genes in COVID-19 [6,7].

Several studies focusing on the viral mechanisms of SARS-CoV-2 infection have pointed to the importance of the surface receptor for the viral spike 1 protein (S1) of the angiotensin-converting enzyme 2 (ACE2) and transmembrane protease serine-2 (TMPRSS2) receptor in COVID-19 [8]. It is known that SARS-CoV-2 can translocate via membrane ACE2 exopeptidase into the host cells, and thus hypomethylation of ACE2 possibly upregulates its expression, enhancing the risk of SARS-CoV-2 infection [9]. Similar results were found in a previous study, where acute respiratory issues were associated with hypomethylation in the ACE2 promoter in blood [10]. Further, a study involving 500 COVID-19 patients showed that the involvement of the ACE2 gene depends on multiple individual variables such as sex, age, body mass index, smoking, and the presence of comorbidities, confirming hypomethylation in the ACE2 gene’s promoter [11].

A growing healthcare problem associated with COVID-19 is the presence of long-lasting symptoms after the infection. The presence of a long-lasting symptomatology after COVID-19 has been called long COVID [12] or post-COVID-19 condition [13]. More than 100 long-lasting post-COVID-19 symptoms affecting respiratory, cardiovascular, immune, neurological, gastrointestinal, or musculoskeletal systems can be attributed to SARS-CoV-2 infection [14]. The Global Burden of Disease Long COVID study (which included 1.2 million of COVID-19 survivors) found that around 15% of individuals who had surpassed a SARS-CoV-2 infection experience post-COVID-19 symptoms up to one year after [15]. Thus, a recent meta-analysis found that up to 25–30% of patients reported post-COVID-19 symptoms two years after infection [16].

The underlying mechanisms explaining the development of post-COVID-19 symptomatology are still unknown and epigenetics have emerged as one potential crucial factor in elucidating them [17]. The fact that methylation changes identified during the acute COVID-19 phase persist one year after acute infection [18] can open a door for exploring an epigenetic relevance in the development of long-lasting post-COVID-19 symptoms. Thus, Nikesjö et al. described a specific DNA methylation signature in ten COVID-19 survivors suffering from post-COVID-19 symptoms lasting up to 10 months [19]. However, the processes by which epigenetics might fine tune the presence of long-lasting post-COVID-19 symptoms remain a major challenge, particularly in humans.

Therefore, the aim of this study was to investigate if DNA methylation pattern of the ACE2 promoter is associated with the presence of post-COVID-19 symptomatology in a cohort of individual who were hospitalized due to an acute SARS-CoV-2 infection.

## 2. Methods

### 2.1. Participants

A cohort of individuals who were hospitalized due to an acute SARS-CoV-2 infection during the first wave of the COVID-19 pandemic (March–May 2020) at four different urban hospitals in Madrid (Spain) were invited to participate in this study. To be included, a diagnosis of SARS-CoV-2 infection at hospital admission should have been confirmed by reverse transcription-polymerase chain reaction (RT-PCR) assay of nasopharyngeal and oral swab sample as well as clinical/radiological findings. The study was approved by the Ethics Committees of all involved institutions and hospitals (URJC0907202015920; HUFA 20/126; HCSC20/495E, HSO25112020; HUIL/092-20). All participants provided their written informed consent prior to the collection of any data.

### 2.2. Genome DNA Collection

Unstimulated whole saliva samples were collected into collection tubes according to standardized procedures: 1, patients were seated; 2, data collection was always conducted during the morning; and 3, patients were asked not to eat, drink, or chew gum for 1 h before sample collection. Saliva samples were centrifuged at 3000 rpm for 15 min to obtain the cell sediment and a self-collection procedure was carried out immediately afterwards and the samples were stored at −20 °C until the analysis. Saliva was used instead of whole blood because it is non-invasive, stress-free, and ethically suitable assessment method.

Genomic DNA was extracted from 500 mL of saliva using a MagMAX™ DNA Multi-Sample Ultra 2.0 Kit (Thermo Fisher Scientific Inc., Hemel Hempstead, Hertfordshire, UK) according to the manufacturer’s protocol. We automatically extracted DNA using the King Fisher Flex purification robot (Thermo Fisher). The resulting DNA was assessed for purity and concentration using Quant-iT™ PicoGreen™ dsDNA reagent” (Thermo Fisher, Waltham, MA, USA).

### 2.3. Differentially Methylation Profiling

**Figure 1:**
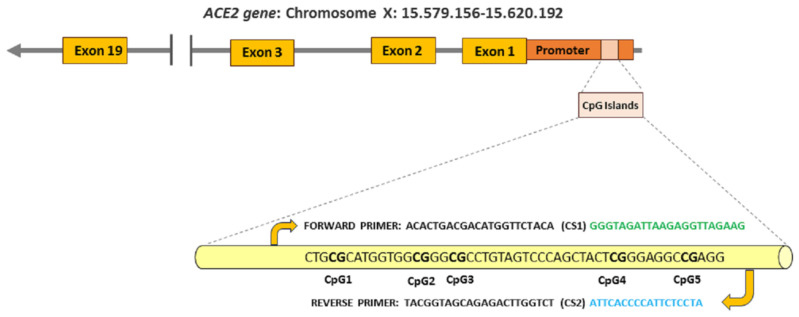
The CpG island sequence in the promoter region of human ACE2. A total of 5 CpG sites were analyzed. CpG sites are in bold. The primers used for DNA methylation sequencing are shown. Forward primer is shown in green color and reverse primer is shown in blue color. Both primers were fused to a 5′-tail comprising sequences (CS1 and CS2) necessary for library preparation and sequencing reactions.

Genomic DNA was bisulfite converted using the Epitech Fast 96 Bisulfite Kit (Cat nº 50959720, Werfen España, Barcelona) following the manufacturer’s instructions. As a measure of successful conversion, the overall percentages of non-cytosine-phosphate-guanine (CpG) dinucleotides methylation varied from 0.03 to 0.06% among all loci and samples. Analyses of ACE2 promoter methylation were amplified using tailed oligos, i.e., a unique amplicon-specific part, fused to a 5′-tail comprising sequences necessary for library preparation and sequencing reactions. A web-based program (http://www.urogene.org/methprimer, accessed on 1 February 2024) was used to identify CpG sites in the ACE2 promoter. Accordingly, five CpG sites of interest (CpG1, CpG2, CpG3, CpG4, CpG5) within the ACE2 promoter were selected according to the general rules and advice for primer design that were previously described [20,21] (Figure 1). The scores (percentage) were calculated by the PyroMark Assay Design, version 2.0.1.15 (Qiagen GmbH).

Analysis was performed using real time PCR with TB Green Premix Ex Taq II mastermix (Takara, France). Following ACE2-specific amplification, amplification products were purified from agarose gels, titrated, and diluted for further processing. NGS libraries were made using a collection of Illumina-compatible PCR primers including a 10 bp barcode identifier (MID) which was used to identify each sample within the pool. The products of this second amplification were evaluated using Bioanalyzer chips (Agilent, Santa Clara, CA, USA), titrated, and pooled, followed by additional bead-based purification and quantification. Finally, samples were subjected to Illumina sequencing in MiSeq (2 × 250 reads) (Illumina, Cambridge, UK).

The sequencing run yielded over 840,000 filtered, quality reads, an average of about 1800 reads per amplicon per sample (range 500 to 5000).

Bisulfite conversion, amplification of target sequences and sequencing were carried out at Fundación Parque Científico de Madrid (FPCM), c/Faraday 7, Madrid, Spain). Reads obtained were filtered and sorted according to their MID and the reference sequence. Alignments and calculation of the percentage methylation were subsequently performed using the freely available software Bismark (version 22.3). The percentage of methylation per sample within each CpG was calculated as the percentage C/C+T and used in the correlation analyses, the mean value of all CpG dinucleotides per amplicon was calculated to represent the methylation value of a particular locus. We analyzed the methylation percentage (%) of each position (CpG1, CpG2, CpG3, CpG4, CpG5) separately for the analysis

### 2.4. Collection Data

Data related to hospitalization due to COVID-19 were collected from hospital medical records: previous medical conditions, admission to intensive care unit (ICU), hospitalization stay (days).

Participants were scheduled for a face-to-face interview conducted by a healthcare professional. We used the definition proposed by Soriano et al. [13]: “post-COVID-19 condition occurs in people with a history of probable or confirmed SARS-CoV-2 infection, usually three months from the onset of infection, with symptoms that last for at least two months and cannot be explained by an alternative medical diagnosis. Common symptoms include, but are not limited to, fatigue, shortness of breath, and cognitive dysfunction, and generally have an impact on everyday functioning” [13]. Accordingly, patients were specifically asked to report the presence of any particular symptom that appeared in the following three months after their hospitalization due to SARS-CoV-2 infection and if that particular symptom still persisted at the time of the appointment. A predetermined list of symptoms (e.g., fatigue, dyspnea, brain fog, memory loss, anosmia, ageusia, hair loss, skin rashes, concentration loss, pain) was systematically used, although participants were free to report any symptom that they suffered from and attributed to the infection.

### 2.5. Statistical Analysis

Data were collected with STATA 16.1 and processed using Python’s library pandas 0.25.3. Mean and standard deviation (SD) are presented for quantitative data and number of cases (percentages) are presented for categorical data. Differences in methylation percentages (%) according to the presence/absence of post-COVID-19 symptomatology were analyzed with one-way-ANOVA tests. The Shapiro–Wilk test was used to assess the assumption of normality. For all inferences, the level of significance was set at priori 0.05 with *p*-values from all tests being corrected (Holm–Bonferroni correction). 

## 3. Results

From the 330 patients who were hospitalized due to COVID-19 in the four targeted hospitals during the first wave of the pandemic and who were invited to participate during the study period, 51 (15%) were excluded as follows: refused to attend the appointment (n = 30), saliva sample was compromised during methylation analyses (n = 14), and pregnancy (n = 7). Finally, 279 (48.7% female, mean age: 56.4 ± 12.8 years) patients were included in the study.

At the follow-up assessment (mean: 17.8; SD: 5.2 months after hospital discharge), 246 (88.1%) patients exhibited at least one post-COVID-19 symptom (mean number of symptoms per patient: 3.0; SD: 1.9). Dyspnea at exertion (67.3%), fatigue (62.3%), and memory loss (31.2%) were the most prevalent post-COVID-19 symptoms. Other prevalent post-COVID-19 symptoms were concentration loss (15%) and brain fog (14.6%).

For the main analyses, we considered the following post-COVID-19 symptoms: fatigue, dyspnea at rest, dyspnea at exertion, brain fog, memory loss, concentration loss, and gastrointestinal problems. Overall, the analysis did not reveal differences in the methylation of the ACE2 promoter in any of the CpG locations according to the presence or absence of fatigue (Table 1), dyspnea at rest (Table 2), dyspnea at exertion (Table 3), memory loss (Table 4), brain fog (Table 5), concentration loss (Table 6), and gastrointestinal problems (Table 7).

Small differences were identified depending on the presence/absence of some post-COVID-19 symptoms. The most significant difference was that the presence of post-COVID-19 fatigue (*p* = 0.007, Table 1), dyspnea on exertion (*p* = 0.008, Table 3), or concentration loss (*p* = 0.007, Table 6) was more prevalent in females than in males. In addition, a significantly higher proportion of patients reporting post-COVID-19 memory loss suffered from diabetes (*p* = 0.046) or asthma (*p* = 0.014) before the infection.

## 4. Discussion

This study did not find an association between the methylation of the ACE2 promoter and the presence of post-COVID-19 fatigue, dyspnea, cognitive or gastrointestinal problems up to one year and a half after the infection in previously hospitalized COVID-19 survivors.

We observed that up of 90% of our cohort of COVID-19 survivors who were hospitalized during the first wave of the pandemic reported post-COVID-19 symptomatology up to 18 months after hospital discharge because SARS-CoV-2. Previous meta-analyses have reported that 25–30% of COVID-19 survivors exhibit post-COVID-19 symptoms one or two years after an acute SARS-CoV-2 infection [15,16]; thus, our prevalence rate was much higher than that in the published literature. Different features of our cohort of COVID-19 survivors could explain the differences in prevalence rates of post-COVID-19 symptoms. First, the sample included in our study were patients infected with the historical strain (i.e., during the first wave of the pandemic). Current data suggest that the prevalence rate of post-COVID-19 symptoms is higher in patients infected with the historical strain than in those individuals infected with the Alpha, Delta, or Omicron variants [22,23]. Second, all participants in our study had been infected and developed post-COVID-19 symptoms before vaccination. Evidence supports that vaccination is able to decrease the risk of post-COVID-19 symptomatology if administered before SARS-CoV-2 infection and before the development of post-COVID-19 symptoms, but its effects on those with ongoing symptomatology is still not clear [24]. Third, the current study included a cohort of previously hospitalized patients with, therefore, moderate to severe COVID-19. Although it has been suggested that hospitalized and non-hospitalized patients develop post-COVID-19 symptoms, a meta-analysis has found that COVID-19 survivors who had been hospitalized are at a higher risk of suffering from some post-COVID-19 symptoms such as dyspnea or pain than COVID-19 survivors who are not hospitalized [25].

It seems that the post-COVID-19 condition has multifactorial and multiple mechanisms, e.g., viral persistence, long-lasting inflammation, endothelial dysfunction, reactivation latent infections, immune system dysregulation, and alteration in gut microbiota have been proposed [17]. Our study did not find an association between methylation of ACE2 promoter and the presence of long-lasting post-COVID-19 fatigue, dyspnea, cognitive or gastrointestinal problems. The present results are contrary to those found by Nikesjö et al. who described a specific DNA methylation signature in ten COVID-19 survivors with post-COVID-19 symptomatology 10 months after the acute infection [19]. Similarly, Balnis et al. identified a hypermethylation pattern in interferon-related genes and a hypomethylation pattern in inflammatory-related genes not only at the acute COVID-19 phase [7] but also one year after the infection [18] in a sample of 15 patients. Differences in DNA methylation techniques and specific gene promoters could explain discrepancies among the studies. It is possible that DNA methylation of gene promoters related to the pro-inflammatory response associated with SARS-CoV-2 could be revealed to have some associations with post-COVID-19 symptomatology. In addition, the small sample size and the lack of a comparative group of COVID-19 survivors without post-COVID-19 symptoms in previously published studies also limit their comparability with the current one.

An important topic to consider is that no timeframe can currently be made for DNA methylation, since this is variable [26]. In fact, no longitudinal studies have investigated the possible variations of DNA methylation throughout time. It is possible that SARS-CoV-2 can lead to DNA methylation changes in some genes at the acute phase of the infection but these changes reverse with time. The fact that DNA methylation alterations are reversible opens the possibility of using DNA methylation or demethylation as targets for therapeutic treatments [27].

Finally, the results of the current study should be analyzed considering its potential limitations. First, we included a cohort of patients who were hospitalized due to COVID-19 during the first wave of the pandemic, when the historical SARS-CoV-2 strain was predominant; hence, an extrapolation of our results should be performed with caution. Second, the cross-sectional design did not permit to identify the longitudinal evolution of DNA methylation alterations and the fluctuating nature of these changes. Third, the current study focused solely on DNA methylation changes in the ACE2 promoter. Population-based studies that include whole DNA methylation analyses could help to identify epigenetic changes associated with post-COVID-19 symptomatology.

## 5. Conclusions

This study did not find an association between methylation of ACE2 promoter and the presence of post-COVID-19 fatigue, dyspnea, cognitive or gastrointestinal problems up to one and a half years after an acute SARS-CoV-2 infection in a cohort of COVID-19 survivors who required hospitalization during the first wave of the outbreak.

## Figures and Tables

**Table 1:** Demographic, clinical, and methylation percentages in COVID-19 patients with or without post-COVID-19 fatigue.

	Post-COVID-19 Fatigue (n = 174)	No Post-COVID-19 Fatigue (n = 105)	*p* Value
Age, mean (SD), years	57.0 (12.5)	55.7 (13.1)	0.423
Gender, male/female (%) *	74 (42.5%)/100 (57.5%)	69 (65.7%)/36 (34.3%)	0.007 *
Weight, mean (SD), kg	81.5 (18.0)	80.5 (15.0)	0.675
Height, mean (SD), cm	166.5 (11.5)	169.0 (9.2)	0.679
Number of medical conditions	1.3 (1.0)	1.1 (1.0)	0.523
Pre-existing medical conditions, n (%)			
Hypertension	58 (33.3%)	37 (35.25%)	0.791
Diabetes	21 (12.1%)	8 (7.6%)	0.264
Cardiovascular Diseases	12 (6.9%)	8 (7.6%)	0.829
Asthma	20 (11.5%)	11 (10.5%)	0.805
Obesity	60 (34.5%)	25 (23.8%)	0.118
Chronic Obstructive Pulmonary Disease	3 (1.7%)	2 (1.9%)	0.913
Number of COVID-19-onset symptoms, mean (SD)	3.25 (1.0)	3.1 (1.0)	0.218
Days at hospital, mean (SD)	7.0 (5.8)	8.6 (10.0)	0.136
Intensive Care Unit (ICU) admission			
Yes/No, n (%)	8 (4.5%)/166 (95.5%)	2 (2%)/103 (98%)	0.326
CpG1 methylation (%)	93.3 (4.0)	93.7 (3.2)	0.335
CpG2 methylation (%)	40.4 (7.4)	39.4 (7.3)	0.259
CpG3 methylation (%)	43.6 (9.0)	42.8 (8.1)	0.437
CpG4 methylation (%)	45.5 (8.0)	45.6 (7.8)	0.937
CpG5 methylation (%)	0.6 (0.3)	0.6 (0.4)	0.804

n: number; SD: standard deviation; * Statistically significant differences between groups (*p* < 0.05).

**Table 2:** Demographic, clinical, and methylation percentages in COVID-19 patients with or without post-COVID-19 dyspnea at rest.

	Post-COVID-19 Dyspnea at Rest (n = 36)	No Post-COVID-19 Dyspnea at Rest (n = 243)	*p* Value
Age, mean (SD), years	55.0 (15.5)	57.7 (12.4)	0.421
Gender, male/female (%)	11 (30.5%)/25 (69.5%)	132 (64.3%)/111 (45.7%)	0.07
Weight, mean (SD), kg	81.0 (20.0)	81.9 (16.5)	0.933
Height, mean (SD), cm	165.5 (8.7)	168.0 (9.5)	0.501
Number of medical conditions	1.3 (1.0)	1.3 (1.0)	0.724
Pre-existing medical conditions, n (%)			
Hypertension	13 (36.1%)	82 (33.7%)	0.820
Diabetes	7 (19.4%)	22 (9.0%)	0.081
Cardiovascular Diseases	1 (2.8%)	19 (7.8%)	0.291
Asthma	4 (11.1%)	27 (11.1%)	0.636
Obesity	14 (38.9%)	71 (29.2%)	0.327
Chronic Obstructive Pulmonary Disease	1 (2.8%)	4 (1.7%)	0.636
Number of COVID-19-onset symptoms, mean (SD)	3.4 (1.0)	3.2 (1.0)	0.125
Days at hospital, mean (SD)	8.7 (5.8)	7.9 (9.0)	0.617
Intensive Care Unit (ICU) admission			
Yes/No, n (%)	1 (2.8%)/35 (97.2%)	9 (3.7%)/107 (96.3%)	0.623
CpG1 methylation (%)	93.0 (4.5)	93.5 (3.6)	0.350
CpG2 methylation (%)	37.9 (8.3)	40.3 (7.2)	0.061
CpG3 methylation (%)	40.7 (10.0)	43.7 (8.4)	0.054
CpG4 methylation (%)	44.3 (9.7)	45.7 (7.6)	0.322
CpG5 methylation (%)	0.6 (0.25)	0.6 (0.4)	0.623

n: number; SD: standard deviation.

**Table 3:** Demographic, clinical, and methylation pe percentages in COVID-19 patients with or without post-COVID-19 dyspnea on exertion.

	Post-COVID-19 Dyspnea on Exertion (n = 188)	No Post-COVID-19 Dyspnea Exertion (n = 91)	*p* Value
Age, mean (SD), years	56.5 (13.2)	56.5 (12.0)	0.997
Gender, male/female (%) *	82 (43.6%)/106 (56.4%)	61 (67.0%)/30 (33.0%)	0.008 *
Weight, mean (SD), kg	81.0 (17.5)	81.1 (15.0)	0.967
Height, mean (SD), cm	166.5 (9.5)	169.0 (9.6)	0.282
Number of medical conditions	1.3 (1.0)	1.1 (1.0)	0.205
Pre-existing medical conditions, n (%)			
Hypertension	62 (33.0%)	33 (36.25%)	0.659
Diabetes	20 (10.6%)	9 (9.9%)	0.855
Cardiovascular Diseases	14 (7.5%)	6 (6.6%)	0.802
Asthma	24 (12.8%)	7 (7.7%)	0.233
Obesity	64 (34.0%)	21 (23.1%)	0.120
Chronic Obstructive Pulmonary Disease	3 (1.6%)	2 (2.2%)	0.727
Number of COVID-19-onset symptoms, mean (SD)	3.2 (1.0)	3.25 (1.0)	0.637
Days at hospital, mean (SD)	8.2 (9.6)	7.6 (6.2)	0.608
Intensive Care Unit (ICU) admission			
Yes/No, n (%)	7 (3.7%)/181 (96.3%)	3 (3.3%)/88 (96.7%)	0.538
CpG1 methylation (%)	93.2 (4.2)	94.1 (2.5)	0.052
CpG2 methylation (%)	40.2 (7.6)	39.7 (6.9)	0.578
CpG3 methylation (%)	43.4 (8.9)	43.0 (8.5)	0.681
CpG4 methylation (%)	45.7 (8.1)	45.4 (7.5)	0.742
CpG5 methylation (%)	0.6 (0.35)	0.6 (0.35)	0.517

n: number; SD: standard deviation; * Statistically significant differences between groups (*p* < 0.05).

**Table 4:** Demographic, clinical, and methylation percentages in COVID-19 patients with or without post-COVID-19 memory loss.

	Post-COVID-19 Memory Loss (n = 87)	No Post-COVID-19 Memory Loss (n = 192)	*p* Value
Age, mean (SD), years	57.9 (12.3)	55.8 (13.0)	0.204
Gender, male/female (%)	8 (44.7%)/49 (56.3%)	107 (54.7%)/87 (45.3%)	0.222
Weight, mean (SD), kg	81.2 (16.9)	81.0 (16.8)	0.867
Height, mean (SD), cm	166.7 (9.5)	168.0 (9.5)	0.469
Number of medical conditions	1.45 (1.0)	1.2 (1.0)	0.07
Pre-existing medical conditions, n (%)			
Hypertension	35 (40.2%)	60 (31.25%)	0.233
Diabetes *	14 (16.1%)	15 (7.8%)	0.046 *
Cardiovascular Diseases	6 (6.9%)	14 (7.3%)	0.909
Asthma *	16 (18.4%)	15 (7.8%)	0.014 *
Obesity	24 (27.6%)	61 (31.8%)	0.557
Chronic Obstructive Pulmonary Disease	0 (0.0%)	5 (2.6%)	0.132
Number of COVID-19-onset symptoms, mean (SD) *	3.4 (0.8)	3.1 (1.1)	0.04 *
Days at hospital, mean (SD)	9.1 (12.3)	7.5 (6.4)	0.159
Intensive Care Unit (ICU) admission			
Yes/No, n (%)	4 (4.6%)/83 (95.4%)	6 (3.1%)/186 (96.9%)	0.09
CpG1 methylation (%)	93.1 (4.9)	93.6 (3.1)	0.263
CpG2 methylation (%)	41.1 (7.1)	39.5 (7.4)	0.096
CpG3 methylation (%)	44.6 (8.3)	42.7 (8.7)	0.087
CpG4 methylation (%)	46.5 (7.8)	45.1 (7.9)	0.177
CpG5 methylation (%)	0.6 (0.25)	0.6 (0.4)	0.086

n: number; SD: standard deviation; * Statistically significant differences between groups (*p* < 0.05).

**Table 5:** Demographic, clinical, and methylation percentages in COVID-19 patients with or without post-COVID-19 brain fog.

	Post-COVID-19 Brain Fog (n = 41)	No Post-COVID-19 Brain Fog (n = 238)	*p* Value
Age, mean (SD), years	55.1 (12.8)	56.7 (12.8)	0.459
Gender, male/female (%)	17 (41.5%)/24 (58.5%)	126 (52.9%)/112 (47.1%)	0.331
Weight, mean (SD), kg	81.6 (19.0)	81.0 (16.3)	0.805
Height, mean (SD), cm	166.5 (9.9)	167.5 (9.5)	0.580
Number of medical conditions	1.35 (1.0)	1.3 (1.0)	0.331
Pre-existing medical conditions, n (%)			
Hypertension	11 (26.83%)	84 (35.3%)	0.390
Diabetes	6 (14.6%)	23 (9.7%)	0.361
Cardiovascular Diseases	1 (2.5%)	19 (8.0%)	0.221
Asthma	7 (17.1%)	24 (10.1%)	0.215
Obesity	14 (34.1%)	71 (29.8%)	0.643
Chronic Obstructive Pulmonary Disease	0 (0.0%)	5 (2.1%)	0.353
Number of COVID-19-onset symptoms, mean (SD)	3.4 (0.9)	3.15 (1.0)	0.142
Days at hospital, mean (SD)	7.5 (7.0)	8.1 (9.0)	0.673
Intensive Care Unit (ICU) admission			
Yes/No, n (%)	2 (4.9%)/39 (95.1%)	8 (3.3%)/230 (96.7%)	0.657
CpG1 methylation (%)	93.7 (4.4)	93.4 (3.7)	0.748
CpG2 methylation (%)	41.4 (6.6)	39.8 (7.5)	0.197
CpG3 methylation (%)	44.6 (8.1)	43.1 (8.8)	0.306
CpG4 methylation (%)	46.7 (7.5)	45.4 (8.0)	0.319
CpG5 methylation (%)	0.6 (0.4)	0.65 (0.35)	0.612

n: number; SD: standard deviation.

**Table 6:** Demographic, clinical, and methylation percentages in COVID-19 patients with or without post-COVID-19 concentration loss.

	Post-COVID-19 Concentration Loss (n = 42)	No Post-COVID-19 Concentration Loss (n = 237)	*p* Value
Age, mean (SD), years	54.5 (12.4)	57.0 (12.9)	0.311
Gender, male/female (%) *	16 (28.1%)/26 (61.9%)	127 (54.6%)/110 (46.4%)	0.007 *
Weight, mean (SD), kg	80.7 (16.4)	81.0 (16.9)	0.898
Height, mean (SD), cm	165.5 (9.75)	167.0 (9.5)	0.202
Number of medical conditions	1.3 (1.0)	1.3 (1.0)	0.989
Pre-existing medical conditions, n (%)			
Hypertension	14 (33.3%)	81 (34.2%)	0.931
Diabetes	2 (4.8%)	27 (11.4%)	0.219
Cardiovascular Diseases	2 (4.8%)	18 (7.6%)	0.525
Asthma	4 (9.5%)	27 (11.4%)	0.737
Obesity	19 (45.2%)	66 (27.8%)	0.06
Chronic Obstructive Pulmonary Disease	0 (0.0%)	5 (2.1%)	0.346
Number of COVID-19-onset symptoms, mean (SD)	3.3 (1.1)	3.2 (1.0)	0.554
Days at hospital, mean (SD)	8.5 (7.7)	7.9 (8.8)	0.708
Intensive Care Unit (ICU) admission			
Yes/No, n (%)	2 (4.7%)/40 (95.3%)	8 (3.4%)/229 (94.6%)	0.657
CpG1 methylation (%)	93.1 (4.5)	93.5 (3.6)	0.497
CpG2 methylation (%)	38.9 (7.6)	40.1 (7.3)	0.292
CpG3 methylation (%)	42.2 (8.6)	43.5 (8.7)	0.368
CpG4 methylation (%)	43.5 (7.6)	45.9 (8.0)	0.066
CpG5 methylation (%)	0.6 (0.3)	0.6 (0.4)	0.506

n: number; SD: standard deviation; * Statistically significant differences between groups (*p* < 0.05).

**Table 7:** Demographic, clinical, and methylation percentages in COVID-19 patients with or without post-COVID-19 gastrointestinal symptomatology.

	Post-COVID-19 Gastrointestinal Symptoms (n = 25)	No Post-COVID-19 Gastrointestinal Symptoms (n = 254)	*p* Value
Age, mean (SD), years	55.0 (14.1)	56.6 (12.7)	0.536
Gender, male/female (%)	11 (44.0%)/14 (56.0%)	132 (52.0%)/122 (48.0%)	0.586
Weight, mean (SD), kg	81.2 (21.2)	81.0 (16.4)	0.947
Height, mean (SD), cm	168.5 (12.7)	167.5 (9.2)	0.475
Number of medical conditions	1.2 (1.0)	1.3 (1.0)	0.523
Pre-existing medical conditions, n (%)			
Hypertension	10 (40.0%)	85 (33.5%)	0.593
Diabetes	1 (4.0%)	28 (11.0%)	0.299
Cardiovascular Diseases	1 (4.0%)	19 (7.5%)	0.535
Asthma	3 (12.0%)	28 (11.0%)	0.889
Obesity	50 (20.0%)	80 (31.5%)	0.320
Chronic Obstructive Pulmonary Disease	1 (4.0%)	4 (1.6%)	0.387
Number of COVID-19-onset symptoms, mean (SD)	3.35 (0.8)	3.2 (1.0)	0.408
Days at hospital, mean (SD)	5.7 (2.5)	8.2 (9.0)	0.168
Intensive Care Unit (ICU) admission			
Yes/No, n (%)	0 (0.0%)/25 (100%)	10 (3.9%)/244 (96.1%)	0.412
CpG1 methylation (%)	91.3 (6.0)	93.7 (3.4)	0.002
CpG2 methylation (%)	40.5 (8.0)	40.0 (7.3)	0.711
CpG3 methylation (%)	42.2 (9.2)	43.4 (8.6)	0.527
CpG4 methylation (%)	44.3 (9.5)	45.7 (7.7)	0.411
CpG5 methylation (%)	0.55 (0.45)	0.6 (0.35)	0.295

n: number; SD: standard deviation.

## Data Availability

All data are presented in the text of the paper and are available on appropriate requirement from the corresponding author.

## References

[1] Deans C., Maggert K.A. (2015). What do you mean, “epigenetic”?. Genetics.

[2] Capp J.P. (2021). Interplay between genetic, epigenetic, and gene expression variability: Considering complexity in evolvability. Evol. Appl..

[3] Mantovani A., Netea M.G. (2020). Trained innate immunity, epigenetics, and COVID-19. N. Engl. J. Med..

[4] Behura A., Naik L., Patel S., Das M., Kumar A., Mishra A., Nayak D.K., Manna D., Mishra A., Dhiman R. (2023). Involvement of epigenetics in affecting host immunity during SARS-CoV-2 infection. Biochim. Biophys. Acta Mol. Basis Dis..

[5] Moore L.D., Le T., Fan G. (2013). DNA methylation and its basic function. Neuropsychopharmacology.

[6] Dey A., Vaishak K., Deka D., Radhakrishnan A.K., Paul S., Shanmugam P., Daniel A.P., Pathak S., Duttaroy A.K., Banerjee A. (2023). Epigenetic perspectives associated with COVID-19 infection and related cytokine storm: An updated review. Infection.

[7] Balnis J., Madrid A., Hogan K.J., Drake L.A., Chieng H.C., Tiwari A., Vincent C.E., Chopra A., Vincent P.A., Robek M.D. (2021). Blood DNA Methylation and COVID-19 outcomes. Clin. Epigenet..

[8] Singh H.O., Choudhari R., Nema V., Khan A.A. (2021). ACE2 and TMPRSS2 polymorphisms in various diseases with special reference to its impact on COVID-19 disease. Microb. Pathog..

[9] Faramarzi A., Safaralizadeh R., Dastmalchi N., Teimourian S. (2022). Epigenetic-related effects of COVID-19 on human cells. Infect. Disord. Drug Targets.

[10] Najafipour R., Mohammadi D., Momeni M., Moghbelinejad S. (2022). ACE-2 Expression and methylation pattern in bronchoalveolar lavage fluid and bloods of Iranian ARDS COVID-19 patients. Int. J. Mol. Cell Med..

[11] Daniel G., Paola A.R., Nancy G., Fernando S.O., Beatriz A., Zulema R., Julieth A., Claudia C., Adriana R. (2022). Epigenetic mechanisms and host factors impact ACE2 gene expression: Implications in COVID-19 susceptibility. Infect. Genet. Evol..

[12] Fernández-de-las-Peñas C. (2022). Long COVID: Current definition. Infection.

[13] Soriano J.B., Murthy S., Marshall J.C., Relan P., Diaz J.V., WHO Clinical Case Definition Working Group on Post-COVID-19 Condition (2022). A clinical case definition of post-COVID-19 condition by a Delphi consensus. Lancet Infect. Dis..

[14] Hayes L.D., Ingram J., Sculthorpe N.F. (2021). More Than 100 Persistent Symptoms of SARS-CoV-2 (Long COVID): A scoping review. Front. Med..

[15] Wulf Hanson S., Abbafati C., Aerts J.G., Al-Aly Z., Ashbaugh C., Ballouz T., Blyuss O., Bobkova P., Bonsel G., Global Burden of Disease Long COVID Collaborators (2022). Estimated global proportions of individuals with persistent fatigue, cognitive, and respiratory symptom clusters following symptomatic COVID-19 in 2020 and 2021. JAMA.

[16] Fernández-de-las-Peñas C., Notarte K.I., Macasaet R., Velasco J.V., Catahay J.A., Therese Ver A., Chung W., Valera-Calero J.A., Navarro-Santana M. (2024). Persistence of post-COVID symptoms in the general population two years after SARS-CoV-2 infection: A systematic review and meta-analysis. J. Infect..

[17] Fernández-de-las-Peñas C., Raveendran A.V., Giordano R., Arendt-Nielsen L. (2023). Long COVID or post-COVID-19 condition: Past, present and future research directions. Microorganisms.

[18] Balnis J., Madrid A., Hogan K.J., Drake L.A., Adhikari A., Vancavage R., Singer H.A., Alisch R.S., Jaitovich A. (2023). Whole-Genome methylation sequencing reveals that COVID-19-induced epigenetic dysregulation remains 1 year after hospital discharge. Am. J. Respir. Cell Mol. Biol..

[19] Nikesjö F., Sayyab S., Karlsson L., Apostolou E., Rosén A., Hedman K., Lerm M. (2022). Defining post-acute COVID-19 syndrome (PACS) by an epigenetic biosignature in peripheral blood mononuclear cells. Clin. Epigenet..

[20] Mikeska T., Felsberg J., Hewitt C.A., Dobrovic A. (2011). Analysing DNA methylation using bisulphite pyrosequencing. Methods Mol. Biol..

[21] Fan R., Mao S.Q., Gu T.L., Zhong F.D., Gong M.L., Hao L.M., Yin F.Y., Dong C.Z., Zhang L.N. (2017). Preliminary analysis of the association between methylation of the ACE2 promoter and essential hypertension. Mol. Med. Rep..

[22] Fernández-de-las-Peñas C., Notarte K.I., Peligro P.J., Velasco J.V., Ocampo M.J., Henry B.M., Arendt-Nielsen L., Torres-Macho J., Plaza-Manzano G. (2022). Long-COVID symptoms in individuals infected with different SARS-CoV-2 variants of concern: A systematic review of the literature. Viruses.

[23] Du M., Ma Y., Deng J., Liu M., Liu J. (2022). Comparison of long COVID-19 caused by different SARS-CoV-2 strains: A systematic review and meta-analysis. Int. J. Environ. Res. Public Health.

[24] Watanabe A., Iwagami M., Yasuhara J., Takagi H., Kuno T. (2023). Protective effect of COVID-19 vaccination against long COVID syndrome: A systematic review and meta-analysis. Vaccine.

[25] Yuan N., Lv Z.H., Sun C.R., Wen Y.Y., Tao T.Y., Qian D., Tao F.P., Yu J.H. (2023). Post-acute COVID-19 symptom risk in hospitalized and non-hospitalized COVID-19 survivors: A systematic review and meta-analysis. Front. Public Health.

[26] Møller Johansen L., Gerra M.C., Arendt-Nielsen L. (2021). Time course of DNA methylation in pain conditions: From experimental models to humans. Eur. J. Pain.

[27] Tajerian M., Alvarado S., Millecamps M., Vachon P., Crosby C., Bushnell M.C., Szyf M., Stone L.S. (2013). Peripheral nerve injury is associated with chronic, reversible changes in global DNA methylation in the mouse prefrontal cortex. PLoS ONE.

